# The risk of anxiety symptoms in young adult offspring of parents with mental health problems: Findings from the raine study

**DOI:** 10.1192/j.eurpsy.2021.1641

**Published:** 2021-08-13

**Authors:** G. Ayano, A. Lin, K. Betts, R. Tait, B. Dachew, R. Alati

**Affiliations:** 1 School Of Public Health, Curtin University, Perth, Australia; 2 Telethon Kids Institute, Universityu of Wetsren Austrlia, Perth, Australia; 3 National Drug Research Institute, Faculty Of Health Sciences, Curtin University, Perth, Australia

**Keywords:** Anxiety, depression, mental healath problem. offspring, parent

## Abstract

**Introduction:**

Previous research has suggested that offspring of parents with mental health problems, including depression and anxiety, are at an increased risk of developing anxiety disorders. Few studies have investigated this relationship in young adults.

**Objectives:**

To investigate the risk of anxiety symptoms in young adult offspring of parents with mental health problems

**Methods:**

We used data from the 1989-1991 cohort of the Western Australian Pregnancy (Raine) Study, which is a multi-generational birth cohort study following mothers and their offspring from pregnancy to 28 years of age. The Depression, Anxiety, and Stress Scale (DASS) was used to assess maternal anxiety and depression whereas a self-reported questionnaire was used to assess paternal emotional problems. Anxiety symptoms among offspring at age 20 were measured by using the short form of the Depression, Anxiety, and Stress Scale (DASS 21). A multivariable negative binomial regression model was used to quantify the associations.

**Results:**

After adjustment, maternal anxiety [RR 1.60 (95% CI 1.11-2.32)] and paternal emotional problems [RR 1.32 (95%CI 1.03-1.68)] were associated with an increased risk of anxiety in offspring at age 20 years. Conversely, maternal depressive symptoms [RR 1.04 (95%CI 0.84-1.32)] were not associated with an increased risk of anxiety in offspring.

**Conclusions:**

The present study suggests that maternal anxiety and paternal emotional problems were associated with an increased risk of anxiety in young adult offspring. However, maternal depressive symptoms were not associated with an increased risk of anxiety in the offspring. The findings suggest the potential for targeted screening and intervention of anxiety problems in the offspring.

**Disclosure:**

No significant relationships.

